# Metabolic Syndrome and Serum Liver Enzymes in the General Chinese Population

**DOI:** 10.3390/ijerph13020223

**Published:** 2016-02-17

**Authors:** Shuang Chen, Xiaofan Guo, Shasha Yu, Ying Zhou, Zhao Li, Yingxian Sun

**Affiliations:** Department of Cardiology, the First Affiliated Hospital of China Medical University, Shenyang 110000, China; loscs@126.com (S.C.); guoxiaofan1986@foxmail.com (X.G.); yidasasa@foxmail.com (S.Y.); zhouying8111003@126.com (Y.Z.); meilichian@aliyun.com (Z.L.)

**Keywords:** alanine aminotransferase, aspartate aminotransferase, metabolic syndrome, liver enzymes

## Abstract

*Background*: The aim of this study was to evaluate the associations between alanine aminotransferase (ALT) and aspartate aminotransferase (AST) with metabolic syndrome (MetS) in the general Chinese population. *Methods*: This study was a multicenter, cross-sectional study which was conducted in rural areas of China from the 2012 to 2013 Northeast China Rural Cardiovascular Health Study (NCRCHS), and 11,573 adults with complete data were included in our final analysis. Elevated ALT and AST levels were defined as >40 U/L. Serum ALT and AST levels within the reference range were divided into quartiles, and their associations with MetS were evaluated by logistic regressions. *Results*: A total of 7.4% and 3.5% participants had elevated serum ALT and AST levels, respectively. The prevalence of MetS was 37.3% in males and 45.8% in females. After adjusting for potential confounders, we found ALT level elevation, even within the reference range, was independently associated with MetS. The odds ratio (OR) values of MetS in the ALT quartiles 2–4 groups within the reference range were 1.113 (95% CI: 1.019–1.280), 1.375 (95% CI: 1.212–1.560), 1.878 (95% CI: 1.650–2.138) compared with the ALT quartile 1 group, and OR in the elevated ALT group was 3.020 (95% CI: 2.496–3.653). Positive relationship for MetS was also observed in elevated AST group (OR: 1.689, 95% CI: 1.314–2.171), but within the reference range, the AST level was not associated with MetS. *Conclusions*: Serum ALT level, even within the reference range, was significantly associated with MetS. However, only elevated AST levels above 40 U/L was positively associated with MetS. Within the reference range, we did not find a relationship between AST levels and MetS.

## 1. Introduction

Metabolic syndrome (MetS) is a cluster of disorders, including abdominal obesity, elevated blood pressure, elevated blood glucose, hypertriglyceridemia, and reduced high-density lipoprotein cholesterol (HDL-C) [1]. MetS is recognized to be strongly associated with type 2 diabetes mellitus [2,3] and cardiovascular diseases (CVD) [4,5]. As CVD is one of the major causes of death throughout the world, it has been a public health issue for early detection and early countermeasures against MetS, which can prevent the progression of CVD.

Serum alanine aminotransferase (ALT) and aspartate aminotransferase (AST) are the common liver enzymes of liver function tests and well-known markers of liver damage [6]. Among the liver enzymes, ALT is the most specific marker of liver function and is used as an indirect marker of liver inflammation or injury, but AST is the less specific marker because it also exists in other tissues [7]. It is reported that elevated ALT is closely associated with liver fat accumulation [8] and is considered as a marker of nonalcoholic fatty liver disease (NAFLD) [9]. Moreover, it has been suggested that NAFLD is considered to be a hepatic consequence of MetS [10,11,12,13,14]. Data linking elevated ALT and AST levels with MetS was presented in several cross-sectional studies [15,16,17]. Thus, elevated ALT and AST might be risk factors for MetS. However, it has been suggested that ALT still remained within the reference range in many uncomplicated obesity subjects, and normal ALT levels cannot rule out the existence of liver disease [18,19,20]. Until now, scanty studies among the general population have reported the relationship between ALT within the normal range and MetS [12]. Therefore, the ALT and AST levels, including within the reference range, need to be reviewed fully in relation to MetS. Accordingly, the present study aimed to investigate (1) associations between elevated ALT and AST levels with morbidity of MetS in a large-scale Chinese population; and (2) the relationship between normal liver enzyme levels and MetS in this representative population.

## 2. Materials and Methods

### 2.1. Study Population

We conducted a cross-sectional study from July 2012 to August 2013 in rural areas of Liaoning Province, which is called the Northeast China Rural Cardiovascular Health Study (NCRCHS). A representative sample aged ≥35 years was selected to describe the prevalence, incidence, and natural history of cardiovascular risk factors. The study adopted a multi-stage, stratified, random cluster-sampling scheme. In the first stage of sampling, three counties (Zhangwu, Dawa, and Liaoyang County) were randomly selected to represent the south, east, and north of Liaoning province. In the second stage, one town was randomly selected from each county (a total of three towns). In the third stage, 8–10 rural villages were randomly selected from each township. In total, 26 rural villages were finally included. All eligible permanent residents aged ≥35 years from each village were selected for participation (a total of 14,016 participants). 11,956 individuals agreed and completed this cross-sectional study and the response rate was 85.3%. Approval for the NCRCHS was obtained from the Ethics Committee of China Medical University (Shenyang, China, ethical approval number: AF-SDP-07-1, 0-01). All participants provided written informed consent and all procedures were performed in accordance with ethical standards. If the participants were illiterate, their proxies wrote the informed consents for them. In this study, we used data of baseline and only participants with complete data were included, making a final sample size of 11,573 (5357 men and 6216 women).

### 2.2. Data Collection

Data were collected during a single clinic visit by cardiologists and trained nurses using a standard questionnaire by face-to-face interviews. Before the survey was performed, we invited all eligible investigators to attend the organized training. The training contents included the purpose of this study, how to administer the questionnaire, the standard method of measurement, the importance of standardization, and the study procedures. A strict test was evaluated after this training; only those who scored perfectly on the test could become investigators. During data collection, our inspectors had further instructions and support.

Data on demographic characteristics, lifestyle risk factors, and medical history, were obtained by interview with a standardized questionnaire. The questionnaire was designed by statistical experts and clinical specialists. There was a central steering committee with a subcommittee for quality control. The project management office of Liaoning Province will randomly check 5% of the questionnaires, the unqualified questionnaires will be re-investigated again, and if the investigator made the unqualified questionnaire, we will cancel the qualification of this investigator and abandon all of his or her questionnaires. Educational level was divided into primary school or below, middle school, and high school or above. Smoking and alcohol status were assessed by two types of questions, “Have you ever smoked at least one cigarette per day for over six months/Have you ever taken alcohol at least twice a week for over a year?” and “Do you smoke/take alcohol now?” Respondents were defined as current smokers/drinkers (those who answered YES to both questions), former smokers/drinkers (those who answered YES to the first question and NO to the second one), and never smokers/drinkers (those who answered NO to both questions). Physical activity included occupational and leisure-time physical activity. Occupational and leisure-time physical activity were merged and regrouped into the following three categories: (1) low—subjects who reported light levels of both occupational and leisure-time physical activity; (2) moderate—subjects who reported moderate or high levels of either occupational or leisure-time physical activity; and (3) high—subjects who reported a moderate or high level of both occupational and leisure-time physical activity. Family income was classified as ≤5000, 5000–20,000, and >20,000 CNY/year.

### 2.3. Blood Pressure Measurements

According to American Heart Association protocol, blood pressure was measured three times in a sitting position at 2 min intervals after at least 5 min of rest in a quiet room with the use of an automatic electronic sphygmomanometer (HEM-741C; Omron, Tokyo, Japan). Two doctors checked the calibration of the Omron device using a standard mercury sphygmomanometer every month under the British Hypertension Society protocol [21]. The mean of three BP measurements was taken and used in all analyses.

### 2.4. Anthropometric Measurements

Waist circumference (WC) was measured at the minimum circumference between iliac crest and the rib cage in the standing position at the end of normal expiration using a non-elastic tape. The body mass index (BMI) was calculated using the formula weight (kg)/height^2^ (m^2^).

### 2.5. Biochemical Measurements

Fasting (12 h overnight) blood samples were collected by venepuncture in EDTA tubes. Plasma was subsequently separated and frozen at −20 °C within 1 h for testing at a central, certified laboratory after collection. Fasting plasma glucose (FPG), plasma total cholesterol (TC), triglycerides (TG), low-density lipoprotein cholesterol (LDL-C), high-density lipoprotein cholesterol (HDL-C), serum ALT, AST, and other biochemical parameters were analyzed enzymatically on an Olympus AU640 auto analyzer (Olympus, Kobe, Japan). All laboratory equipment was calibrated and blinded duplicate samples were used.

### 2.6. Elevated Liver Enzymes Definition

Elevated serum ALT or AST level was defined as greater than 40 U/L.

### 2.7. Metabolic Syndrome Criteria

Metabolic syndrome was determined according to the unified criteria published in 2009 [22]. Abdominal obesity was defined as elevated WC: ≥85 cm (male), ≥80 cm (female). Elevated TG: ≥150 mg/dL (1.7 mmol/L). Reduced HDL-C: <40 mg/dL or 1.0 mmol/L (male), <50 mg/dL or 1.3 mmol/L (female). SBP ≥130 mmHg, DBP ≥85 mmHg, or taking antihypertensive agents, were defined as hypertension. FPG of 5.6 mmol/L or higher or taking antidiabetic agents were defined as hyperglycemia.

### 2.8. Statistical Analysis

Continuous variables were expressed as mean values and standard deviation (SD), whereas categorical variables were described as frequencies and percentages. Since data were normally distributed, continuous variables were compared between normal ALT and elevated ALT group by using analysis of variance (ANOVA) test. χ^2^-test analyses were used to examine associations between the categorical variables. Quartiles of ALT and AST were created. Logistic regression was used to estimate the odds ratios (ORs) and 95% CIs for MetS after adjustment for age, race, BMI, and lifestyle factors (smoking, drinking, family income, education, and physical activity). Serum ALT and AST levels within the reference range were divided into quartiles so that the numbers of subjects in the four categories were almost equal, and the lowest category was set as reference. A Pearson’s correlation analysis was used to evaluate independent associations between liver enzyme levels and components of Mets. The thresholds for liver enzyme levels were generated by ROC analysis. All statistical analyses were performed using SPSS version 19.0 software (SPSS Inc., Chicago, IL, USA), and *p* < 0.05 indicated statistical significance.

## 3. Results

**Figure 1:**
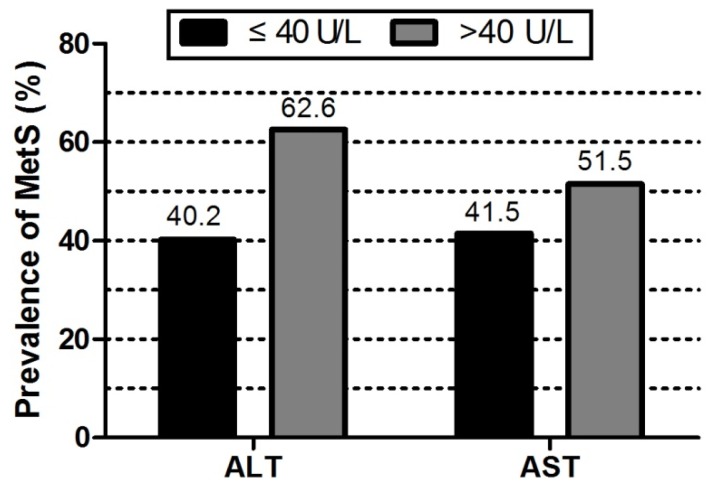
Comparison of the prevalence of metabolic syndrom in both groups of ALT/AST ≤ 40 U/L and ALT/AST > 40 U/L.

Comparisons of characteristics between subjects with and without MetS were shown in Table 1. A total of 11,573 adults (5357 men and 6216 women) aged ≥35 years were included in the study. The prevalence of MetS was 41.9% in general population (37.3% for men and 45.8% for women, separately). Subjects with MetS had statistically significant higher values of SBP, DBP, WC, BMI, TC, TG, LDL-C, FPG, but lower value of HDL-C compared to subjects without MetS (*p* < 0.001). As shown in Figure 1, participants with elevated ALT or AST had statistically significant higher prevalence of MetS compared to the participants with normal liver enzyme levels (*p* < 0.001).

Table 2 and Table 3 showed characteristics of participants stratified by ALT and AST quartiles, respectively. The study population was divided into five groups: one group for elevated ALT/AST (>40 U/L), and four groups within reference range according to ALT/AST quartile. The serum ALT and AST quartile was as follows: <14.0 U/L, 14.0–17.6 U/L, 17.6–23.0 U/L, 23.0–40 U/L for ALT; <17.0 U/L, 17.0–20.0 U/L, 20.0–23.0 U/L, 23.0–40 U/L for AST. Subjects in the highest ALT or AST group were more likely to be men, current smokers and drinkers, had higher blood pressure, WC and BMI, had higher TC, TG, LDL-C, FPG, and higher prevalence of MetS, but had lower HDL-C level than those of subjects in the lowest ALT or AST quartile group.

**Figure 2:**
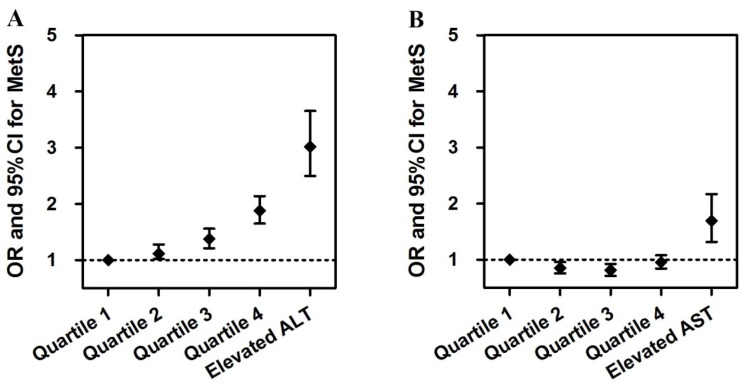
Adjusted odds ratios for having MetS in four age groups, for (**A**) ALT and (**B**) AST.

Table 4 and Figure 2 presented the multiple logistic regression analysis for MetS. The adjusted odds ratios for having MetS, for ALT and AST separately, in the five groups, were depicted in Figure 2. After adjustment for age, sex, race, smoking status, alcohol intake, education degree, physical activity, family income, BMI, and history of CVD, we found that both elevated ALT and elevated AST were associated with higher prevalence of MetS (OR 3.020, 95% CI: 2.496–3.653 for elevated ALT; OR 1.689, 95% CI: 1.314–2.171 for elevated AST). Subjects with elevated liver enzymes tended to exhibit a higher likelihood of having MetS. Even within the reference range, subjects with the highest ALT quartile had a 1.878-times higher risk of MetS than those with the lowest ALT quartile (*p* < 0.001). While in the normal AST level, we did not find a statistically significant relationship between AST level and MetS.

Table 5 showed the Pearson’s correlation coefficients between the liver enzyme levels and other metabolic variables after adjusted for age and sex. The ALT level was positively correlated with the following factors: BMI, WC, SBP, DBP, TG, and FPG; negative correlation was observed for HDL-C. Similarly, the AST level was positively correlated with all the variables listed above, except for HDL-C and FPG.

In addition, we drew the ROC curve according to serum liver enzyme levels and prevalence of MetS. Serum liver enzyme levels when the sum of sensitivity and specificity reached the maximum value was regarded as the optimal cutoff value. The optimal cutoff value of ALT and AST levels were 20.1 U/L and 24.0 U/L, respectively. The sensitivity for diagnosis of MetS, respectively, was 76.8% for ALT and 69.4% for AST, and the specificity, respectively, was 81.4% for ALT and 70.0% for AST.

## 4. Discussion

In this sample of Chinese adults, we investigated independent associations of serum ALT and AST elevation, even within the reference range, with the prevalence of MetS. Our findings showed that participants with higher ALT or AST level were more likely to be obese, have higher blood pressure, TC, TG, LDL-C, FPG level, and have lower HDL-C levels. Subjects had increased prevalence of MetS from the lowest ALT or AST quartile group to the highest quartile group.

Recent epidemiologic studies have reported associations between elevated serum levels of liver enzymes and increased cardiovascular risk. Elevated ALT and gamma-glutamyl transferase (GGT) have been shown to predict CVD in prospective studies [23,24,25]. MetS was suggested to be a risk factor for CVD, and the prevalence had been increasing in developing countries. So the relationships between serum liver enzymes and MetS have drawn significant attention in recent years. We found that elevated serum ALT and AST were significantly associated with increased prevalence of MetS in this Chinese population. Regarding analysis for values above the reference, the associations between elevated ALT or AST and prevalence of MetS had been observed in several different populations, including elderly males [26], obese adults [27], postmenopausal women [28], and even adolescents [29]. Kim HC *et al.* found that the serum ALT level was significantly related to MetS in men but not in women [30]. Previous studies also reported ALT elevation increased the risk for non-hepatic diseases including diabetes mellitus type 2, MetS, CVD, and malignancies [31,32]. Although similar findings have already been reported in middle-aged, urban Chinese men [33], we showed a significant association between elevated liver enzyme levels and MetS in a large-scale general population in China. Among hepatic enzymes, ALT is considered to be the most specific indicator of hepatic diseases and most closely related to liver fat accumulation [34]. In our study, the positive relationship of elevated ALT with MetS was stronger than that of elevated AST. Our data showed that subjects with elevated ALT level had 3.0-fold increased risk of MetS than partners with the lowest ALT quartile group whereas 1.7-folds in elevated AST level. This can be explained by higher specificity of ALT to liver disease. Furthermore, another more important finding of this study was that a statistically significant relationship was observed between serum ALT levels within the normal range and MetS in a level-related manner. These results were in agreement with two Japanese studies [35,36] and a Korean study [37], which found that risk of MetS increased with elevation in serum ALT level within normal range. Previous studies did not evaluate the association between AST levels within the reference range and MetS, but our results indicated that no statistically significant relationship existed with prevalence of MetS and ALT level within normal range.

The most probable explanation for our results of relationship between serum liver enzyme levels and prevalence of MetS is NAFLD. It has been reported that, clinically, the most common etiology of elevated ALT levels is NAFLD [10,38]. NAFLD is a probable explanation for abnormal liver enzyme levels and a cause of asymptomatic elevation of ALT levels [39]. NAFLD is considered to be associated with metabolic disorders, including obesity, hypertension, hyperglycemia, and dyslipidemia, which are components of the MetS [40,41]. Therefore, this supports the notion that NAFLD is considered a hepatic manifestation of MetS [42].

In our study, the optimal cutoff values of serum ALT and AST concentration was 20.1 U/L and 24.0 U/L, separately. The routine screening of liver enzymes and updated cutoff levels of ALT and AST could help early detection of MetS and to arrest the progress of disease. However, more prospective studies are needed to identify the optimal cutoff values of liver enzymes for Chinese people.

Study strengths and limitations: the strengths of this study are its population-based design, large sample size, and the first evaluation of the associations of ALT, AST levels with MetS, both within the normal range and elevated liver enzyme values in the general Chinese population. Several limitations in this study need to be acknowledged. First, because of its cross-sectional design, we were unable to determine whether or not there was a causal association. Thus, the obtained associations in this study should be considered with caution. Second, there are known causes of elevated liver enzyme levels that were not tested in our study, such as chronic viral hepatitis and other illnesses, and the possibility still exists that unmeasured confounders may explain part of the association.

## 5. Conclusions

In summary, these representative data of the Chinese general population suggested that elevated levels of ALT and AST, were independently associated with an increased prevalence of MetS. Moreover, elevated ALT level was more closely associated with MetS than elevated AST levels. In addition to that, only ALT level within reference range had significant relationship with the MetS while AST within normal range was not associated with MetS. However, further epidemiologic investigations using longitudinal designs are necessary to understand associations between serum ALT or AST levels and MetS.

## Figures and Tables

**Table 1:** Baseline characteristics in subjects with and without metabolic syndrome (***n*** = 11,573).

Variables	Mets	Non-Mets	*p*-Value	Total
	(*n* = 4849)	(*n* = 6724)		(*n* = 11,573)
Age, year	55.5 ± 10.2	52.7 ± 10.6	<0.001 *	53.8 ± 10.6
Male, %	2003 (41.3)	3355 (49.9)	<0.001 *	5357 (46.3)
Race (Han), %	4616 (95.2)	6361 (94.6)	0.136	10,968 (94.8)
Current smoking status, %	1508 (31.1)	2589 (38.5)	<0.001 *	4086 (35.3)
Current drinking status, %	975 (20.1)	1647 (24.5)	<0.001 *	2617 (22.6)
Education, %			<0.001 *	
Primary school or below	2643 (54.5)	3127 (46.5)		5763 (49.8)
Middle school	1750 (36.1)	2965 (44.1)		4717 (40.8)
High school or above	456 (9.4)	632 (9.4)		1093 (9.4)
Physical activity, %			<0.001 *	
Low	1673 (34.5)	1768 (26.3)		3441 (29.7)
Moderate	2871 (59.2)	4606 (68.5)		7479 (64.7)
High	305 (6.3)	350 (5.2)		653 (5.6)
Family income, CNY/year, %			0.249	
≤5000	635 (13.1)	807 (12.0)		1441 (12.5)
5000–20,000	2619 (54.0)	3685 (54.8)		6306 (54.5)
>20,000	1595 (32.9)	2232 (33.2)		3826 (33.0)
History of CVD, %	946 (19.5)	800 (11.9)	<0.001 *	1712 (15.1)
SBP, mm Hg	150.6 ± 22.6	135.6 ± 22.0	<0.001 *	141.8 ± 23.5
DBP, mm Hg	86.1 ± 11.6	79.2 ± 11.0	<0.001 *	82.1 ± 11.8
WC, cm	88.5 ± 8.3	78.1 ± 8.4	<0.001 *	82.4 ± 9.8
BMI, kg/m^2^	26.8 ± 3.4	23.4 ± 3.2	<0.001 *	24.8 ± 3.7
TC, mmol/L	5.5 ± 1.2	5.1 ± 1.0	<0.001 *	5.2 ± 1.1
TG, mmol/L	2.3 ± 1.9	1.1 ± 0.8	<0.001 *	1.6 ± 1.5
HDL-C, mmol/L	1.2 ± 0.3	1.5 ± 0.4	<0.001 *	1.4 ± 0.4
LDL-C, mmol/L	3.1 ± 0.9	2.9 ± 0.8	<0.001 *	2.9 ± 0.8
FPG, mmol/L	6.5 ± 2.1	5.5 ± 1.0	<0.001 *	5.9 ± 1.6
ALT, U/L	25.3 ± 18.0	20.4 ± 18.5	<0.001 *	22.5 ± 18.4
AST, U/L	22.6 ± 11.7	21.8 ± 12.2	0.001 **^#^**	22.1 ± 12.0

Notes: Data are expressed as the mean ± SD or as *n* (%). Abbreviations: CNY, China Yuan (1 CNY = 0.161 USD); CVD, cardiovascular disease; SBP, systolic blood pressure; DBP, diastolic blood pressure; WC, waist circumference; BMI, body mass index; TC, total cholesterol; TG, triacylglycerol; HDL-C, high-density lipoprotein cholesterol; LDL-C, low-density lipoprotein cholesterol; FPG, fasting plasma glucose; ALT, alanine aminotransferase; AST, aspartate aminotransferase. * *p* < 0.001, **^#^** *p* < 0.05.

**Table 2:** Characteristics of subjects according to the ALT quartiles (*n* = 11,573).

Variables	Normal ALT				Elevated ALT	*p*-Value
	Quartile 1	Quartile 2	Quartile 3	Quartile 4		
Metabolic syndrome, %	979 (30.9)	780 (36.1)	1149 (42.5)	1376 (52.4)	536 (62.6)	<0.001 *
ALT, U/L	11.4 ± 2.2	15.9 ± 0.9	20.2 ± 1.7	29.5 ± 4.7	65.6 ± 43.4	<0.001 *
Age, year	54.8 ± 11.9	54.3 ± 10.8	54.1 ± 9.8	52.9 ± 9.5	51.0 ± 9.4	<0.001 *
Male, %	1028 (32.2)	864 (39.9)	1348 (49.6)	1554 (58.9)	563 (65.5)	<0.001 *
Race (Han), %	3056 (95.8)	2049 (94.5)	2551 (93.9)	2492 (94.5)	820 (95.3)	0.020 **^#^**
Current smoking status, %	1026 (32.2)	732 (33.8)	994 (36.6)	1005 (38.1)	329 (38.3)	<0.001 *
Current drinking status, %	497 (15.6)	423 (19.5)	681 (25.1)	732 (27.7)	284 (33.0)	<0.001 *
High school or above, %	280 (8.8)	180 (8.3)	257 (9.5)	280 (10.6)	96 (11.2)	<0.001 *
High Physical activity, %	177 (5.5)	128 (5.9)	142 (5.2)	162 (6.1)	44 (5.1)	<0.001 *
Family income >20,000 CNY/year, %	1057 (33.1)	662 (30.5)	893 (32.9)	870 (33.0)	344 (40.0)	<0.001 *
History of CVD, %	474 (15.2)	320 (15.1)	398 (14.9)	410 (15.8)	110 (13.1)	0.440
SBP, mmHg	138.5 ± 23.9	141.6 ± 23.8	142.7 ± 23.6	143.9 ± 22.5	144.8 ± 22.1	<0.001 *
DBP, mmHg	79.7 ± 11.5	81.2 ± 11.8	82.3 ± 11.6	84.0 ± 11.6	85.9 ± 12.0	<0.001 *
WC, cm	78.7 ± 8.9	80.9 ± 9.0	83.1 ± 9.5	85.8 ± 9.7	88.0 ± 10.1	<0.001 *
BMI, kg/m^2^	23.5 ± 3.2	24.4 ± 3.5	25.0 ± 3.5	25.9 ± 3.7	26.7 ± 4.2	<0.001 *
TC, mmol/L	5.1 ± 1.0	5.1 ± 1.1	5.3 ± 1.1	5.4 ± 1.1	5.5 ± 1.3	<0.001 *
TG, mmol/L	1.3 ± 1.0	1.4 ± 1.1	1.6 ± 1.4	2.0 ± 1.8	2.3 ± 2.1	<0.001 *
HDL-C, mmol/L	1.4 ± 0.4	1.4 ± 0.4	1.4 ± 0.4	1.4 ± 0.4	1.3 ± 0.4	<0.001 *
LDL-C, mmol/L	2.8 ± 0.8	2.9 ± 0.8	2.9 ± 0.8	3.0 ± 0.9	3.1 ± 0.9	<0.001 *
FPG, mmol/L	5.7 ± 1.5	5.8 ± 1.7	5.9 ± 1.5	6.1 ± 1.6	6.1 ± 1.8	<0.001 *

Notes: The between cut points are 14.0, 17.6, and 23.0 for normal ALT. Data are expressed as the mean ± SD or as *n* (%). Abbreviations: ALT, alanine aminotransferase; CNY, China Yuan (1 CNY = 0.161 USD); CVD, cardiovascular disease; SBP, systolic blood pressure; DBP, diastolic blood pressure; WC, waist circumference; BMI, body mass index; TC, total cholesterol; TG, triacylglycerol; HDL-C, high-density lipoprotein cholesterol; LDL-C, low-density lipoprotein cholesterol; FPG, fasting plasma glucose. * *p* < 0.001, **^#^** *p* < 0.05.

**Table 3:** Characteristics of subjects according to the AST quartiles (*n* = 11,573).

Variables	Normal AST				Elevated AST	*p*-Value
	Quartile 1	Quartile 2	Quartile 3	Quartile 4		
Metabolic syndrome, %	1384 (41.5)	1150 (39.8)	848 (39.6)	1229 (44.9)	209 (51.5)	<0.001 *
AST, U/L	15.2 ± 1.7	18.9 ± 0.8	21.8 ± 0.9	28.0 ± 4.0	64.6 ± 37.6	<0.001 *
Age, year	52.2 ± 10.7	54.3 ± 10.7	55.0 ± 10.4	54.6 ± 10.3	52.4 ± 10.0	<0.001 *
Male, %	1157 (34.4)	1218 (42.0)	1057 (49.1)	1642 (59.6)	283 (69.5)	<0.001 *
Race (Han), %	3232 (96.2)	2764 (95.3)	2025 (94.0)	2572 (93.4)	375 (92.1)	<0.001 *
Current smoking status, %	1059 (31.5)	999 (34.5)	773 (35.9)	1060 (38.5)	195 (47.9)	<0.001 *
Current drinking status, %	449 (13.4)	565 (19.5)	491 (22.8)	926 (33.6)	186 (45.7)	<0.001 *
High school or above, %	326 (9.7)	260 (9.0)	205 (9.5)	264 (9.6)	38 (9.3)	0.094
High Physical activity, %	199 (5.9)	156 (5.4)	136 (6.3)	146 (5.3)	16 (3.9)	0.001 **^#^**
Family income>20,000 CNY/year, %	1250 (37.2)	958 (33.0)	664 (30.8)	820 (29.8)	134 (32.9)	<0.001 *
History of CVD, %	484 (14.7)	461 (16.2)	312 (14.8)	410 (15.2)	45 (11.4)	0.103
SBP, mm Hg	136.7 ± 22.2	140.7 ± 23.0	144.1 ± 23.9	146.6 ± 22.7	146.4 ± 23.8	<0.001 *
DBP, mm Hg	80.3 ± 11.4	81.4 ± 11.6	82.5 ± 11.7	83.9 ± 11.9	86.2 ± 12.4	<0.001 *
WC, cm	81.3 ± 9.4	81.7 ± 9.4	82.5 ± 10.1	84.1 ± 10.2	84.9 ± 10.4	<0.001 *
BMI, kg/m^2^	24.5 ± 3.4	24.6 ± 3.7	24.7 ± 3.6	25.3 ± 3.8	25.3 ± 4.6	<0.001 *
TC, mmol/L	5.1 ± 1.0	5.2 ± 1.0	5.3 ± 1.1	5.4 ± 1.1	5.4 ± 1.5	<0.001 *
TG, mmol/L	1.5 ± 1.2	1.5 ± 1.2	1.6 ± 1.4	1.8 ± 1.7	2.3 ± 2.1	<0.001 *
HDL-C, mmol/L	1.6 ± 0.6	1.4 ± 0.3	1.4 ± 0.4	1.5 ± 0.4	1.3 ± 0.3	<0.001 *
LDL-C, mmol/L	2.8 ± 0.8	2.9 ± 0.8	3.0 ± 0.8	2.9 ± 1.0	3.0 ± 0.9	<0.001 *
FPG, mmol/L	6.1 ± 2.1	5.8 ± 1.4	5.8 ± 1.4	5.8 ± 1.3	6.2 ± 1.9	<0.001 *

Notes: The between cut points are 17.0, 20.0, and 23.0 for normal AST. Data are expressed as the mean ± SD or as *n* (%). Abbreviations: AST, aspartate aminotransferase; CNY, China Yuan (1 CNY = 0.161 USD); CVD, cardiovascular disease; SBP, systolic blood pressure; DBP, diastolic blood pressure; WC, waist circumference; BMI, body mass index; TC, total cholesterol; TG, triacylglycerol; HDL-C, high-density lipoprotein cholesterol; LDL-C, low-density lipoprotein cholesterol; FPG, fasting plasma glucose. * *p* < 0.001, **^#^** *p* < 0.05.

**Table 4:** Multiple logistic regression for metabolic syndrome.

Aminotransferase Levels	OR	95% CI	*p*-Value
Normal ALT			
Quartile 1	1	——	<0.001 *
Quartile 2	1.113	1.019–1.280	0.049 **^#^**
Quartile 3	1.375	1.212–1.560	<0.001 *
Quartile 4	1.878	1.650–2.138	<0.001 *
Elevated ALT	3.020	2.496–3.653	<0.001 *
Normal AST			
Quartile 1	1	——	<0.001 *
Quartile 2	0.852	0.756–0.959	0.008 **^#^**
Quartile 3	0.813	0.713–0.926	0.002 **^#^**
Quartile 4	0.955	0.844–1.080	0.463
Elevated AST	1.689	1.314–2.171	<0.001 *

Notes: Abbreviations: ALT, alanine aminotransferase; AST, aspartate aminotransferase. Adjusted for age, sex, race, smoking, drinking, education, physical activity, family income, history of CVD, and BMI. * *p* < 0.001, **^#^** *p* < 0.05.

**Table 5:** Correlation between serum liver enzymes and investigated variables after adjustments for age and sex.

Variables	ALT		AST	
	Pearson’s Coefficients	*p*-Value	Pearson’s Coefficients	*p*-Value
BMI	0.178	<0.001 *	0.038	<0.001 *
WC	0.201	<0.001 *	0.071	<0.001 *
SBP	0.047	<0.001 *	0.089	<0.001 *
DBP	0.111	<0.001 *	0.098	<0.001 *
TG	0.153	<0.001 *	0.09	<0.001 *
HDL-C	−0.032	0.001 **^#^**	0.139	0.223
FPG	0.055	<0.001 *	0.001	0.955

Notes: Abbreviations: ALT, alanine aminotransferase; AST, aspartate aminotransferase; BMI, body mass index; WC, waist circumference; SBP, systolic blood pressure; DBP, diastolic blood pressure; TG, triacylglycerol; HDL-C, high-density lipoprotein cholesterol; FPG, fasting plasma glucose. * *p* < 0.001, **^#^** *p* < 0.05.

## Abbreviations

The following abbreviations are used in this manuscript:

CNY: China Yuan
CVD: cardiovascular disease
SBP: systolic blood pressure
DBP: diastolic blood pressure
WC: waist circumference
BMI: body mass index
TC: total cholesterol
TG: triacylglycerol
HDL-C: high-density lipoprotein cholesterol
LDL-C: low-density lipoprotein cholesterol
FPG: fasting plasma glucose
ALT: alanine aminotransferase
AST: aspartate aminotransferase
GGT: gamma-glutamyl transferase
